# Examination of learning ability development through the implementation of the “autonomy-collaboration” learning mode grounded in evidence-based medicine practice

**DOI:** 10.1186/s12909-024-05447-6

**Published:** 2024-06-11

**Authors:** Xiao-Huan Li, Qing Zhang, Chun Li, Ya-Lei Yin, Zhen Yang, Ying Fu, Xiao-Lin Yuan

**Affiliations:** 1 Department of preventive medicine, Affiliated Zhongshan Hospital of Dalian University, Dalian116001, Liaoning China; 2 Department of Central Laboratory, Affiliated Zhongshan Hospital of Dalian University, No.6 of Jiefang Street, Zhongshan District, Dalian, Liaoning, 116001 China

**Keywords:** Autonomy, Collaboration, Evidence-based medicine

## Abstract

**Objective:**

Currently, there are still some shortcomings in EBM education in China.The study aimed to investigate the effectiveness of the novel evidence-based medicine (EBM) learning model of “autonomy-collaboration.”

**Methods:**

A total of 91 undergraduate students majoring in clinical medicine at Zhongshan Clinical College of Dalian University from the 2019 batch were selected as the participants in this study. They were instructed to follow the EBM learning model of “autonomy-collaboration.” Upon completion of the course, questionnaires, records of participants’ sentiments and insights, and evidence-based clinical practice reports were used as indicators to evaluate the effectiveness of the training.

**Results:**

This learning modality effectively enhanced independent learning ability of the students, stimulated their interest in learning, and strengthened the communication between students and teachers, thereby improving the quality of teaching.

**Conclusion:**

The novel EBM learning model of “autonomy-collaboration,” exhibited robust effectiveness in instruction and facilitated the seamless integration of theoretical knowledge with clinical practice. Consequently, its widespread adoption is strongly recommended.

## Introduction

In 2008, the Ministry of Education included evidence-based medicine (EBM) in the *Standard for Undergraduate Medical Education* as a mandatory subject for clinical medicine majors. It was also specified that “the initial ability to apply the principles of EBM to investigate and utilize evidence in response to clinical issues” is one of the skill objectives that undergraduate medical students must achieve [1, 2]. The principle of medical education reform is to prioritize student-centered self-directed learning, changing the traditional passive learning approach. It underscores the importance of collaboration and exploration processes to nurture innovation and practical skills in students, explicitly acknowledging them as the primary participants in the learning experience [3]. EBM is an emerging clinical interdisciplinary field that has developed rapidly in clinical practice over the past 20 years. Its fundamental principle is that healthcare programs and decision-making should be based on the most reliable evidence derived from objective clinical scientific research. In this approach the aim is to develop scientific preventive measures to achieve the goal of preventing disease and improving the quality of life [4]. Clinicians need to have an EBM-oriented mindset in their diagnostic and therapeutic practices to make accurate and reliable clinical decisions by combining the best research evidence, personal clinical experience, and the wishes and values of the patients [5–7]. Traditional teaching methods are teacher-centric, often disregarding student autonomy [8], resulting in ineffective teaching outcomes.

Currently, there are still some shortcomings in EBM education in China. For instance, there is an overemphasis on theory over practice. In addition, teaching methods mainly focus only on knowledge training while neglecting the development of skills. How to improve the effectiveness of EBM education, enhance students’ interest in learning, transform them from passive learners to active learners, and cultivate their innovative and practical abilities is crucial. Therefore, in the current study, we aimed to investigate the implementation and effectiveness of the EBM learning model of “autonomy-collaboration,” in clinical lectures. The goal is to enhance teaching quality, promote teaching reform, and improve independent learning ability of the students.

## Participants and methods

### Participants

A total of 91 students majoring in clinical medicine from the 2019 batch at the Zhongshan Clinical College of Dalian University were enrolled in this study. These students were enrolled in the EBM course during the second semester of the 2021 to 2022 academic year.

### Research methodology

A new modality called “autonomy-collaboration” learning based on evidence-based practice competence was adopted for teaching the EBM course. Evidence-based clinical practice is the conscious, explicit and deliberate use of the best available Evidence to develop treatment plans for individual patients. Its main content is to encourage individual doctors to retrieve, evaluate and use research evidence for clinical practice. The teaching was focused on the students, commencing with the “introduction of cases.” The primary teaching measures used were as follows. During the initial phase of autonomous learning, the teacher presented a case based on the requirements of the EBM syllabus. Cases are practical problems encountered in clinical practice, including etiological, diagnostic, preventive and therapeutic problems. The students then analyzed the case according to the knowledge imparted by the teacher. They identified the specific clinical problem associated with the case, and made clinical decisions based on their understanding of EBM. Subsequently, at the conclusion of the session, students filled out a questionnaire that captured their practical application of the case, along with documenting their insights and sentiments about the learning experience. In the second phase of EBM clinical practice, the students were divided into 10 groups of 9 students each (one group of 10), before the classroom lectures. Following the principles of EBM, students used the PICO (population, intervention, control, and outcome) framework to formulate clinical questions for the case. They then conducted a literature search using a hierarchical principle across different databases and evaluated the quality of the literature found. The hierarchical principle of database retrieval refers to: The selection of databases is based on the idea of solving problems based on evidence. The theoretical selection method is to preferentially select Systems databases. If the company does not have Systems or cannot solve problems, Summaries, Synoposes, Syntheses, and Studie are selected step by step, and once the problem is solved at one step, there is no need to continue searching the next level of database. The quality evaluation of literature results is evaluated from three aspects: the authenticity, importance and applicability of evidence (literature results). The specific content of the assessment varies according to different types of clinical problems. Only the retrieved literature meets the above evaluation requirements can be applied to patients. Through collaborative group discussions, they made preliminary clinical decisions based on the specific issues presented in the cases. Furthermore, the exploration phases encompassed group discussions and questioning, effectively integrating theoretical knowledge with practical application, hence emphasizing skill development. The teacher facilitated a group discussion, and the students could communicate with the teacher if there were any doubts. This process concurrently functioned as a review of theoretical knowledge, with the presentation of cases facilitating a more profound comprehension and practical application of the acquired knowledge. Concluding with a practical report, students utilized their EBM knowledge to formulate clinical decisions regarding the real-life clinical issue, documenting their findings in a written report. Evidence-based clinical decision-making means that students solve practical problems in their cases based on their own theoretical knowledge, combined with the literature they have searched and evaluated its authenticity, importance and applicability.

### Evaluation of teaching effectiveness

After completing the EBM courses, students recorded their learning experiences and feelings. Different students had different feelings, as listed below: Some students provided feedback that through case practice, they realized the significant importance of EBM in current medical decision-making. Secondly, there were requirements for doctors, emphasizing the use of professional skills and experience to practice medicine, requiring continuous learning, summarization, and keeping pace with the times. Some students gained insights, acquiring a lot through learning, such as the steps of evidence retrieval and collection, and importantly, through case learning and practical experience, learning how to identify the value of literature, and so on. When students recorded their personal experiences, they also recorded many skills and problem-solving experiences that were unclear before through this course and case study learning, which were fully reflected in the practical reports of learning cases.

Teaching effectiveness was assessed through the analysis of completed questionnaires, students’ reflections and sentiments concerning the case, and evidence-based clinical practice reports, which served as key indicators in the evaluation process.

#### Questionnaire

After the course, a questionnaire was given to the students, which contained the following statements. (1) The course of study enhanced medical knowledge, stimulated learning motivation, and improved learning autonomy. (2) The EBM course is a comprehensive subject that integrates multiple disciplines and improves the comprehensive utilization of various courses and collaborative skills. (3) I acknowledge the importance of EBM in making medical decisions. (4) Improved the ability to identify and raise questions in clinical practice. (5) Adeptly and accurately identifying the type of issue is critical before proceeding to other steps. (6) I acknowledge the significance of accessing and compiling information. (7) Increased awareness to obtain supporting evidence for any decision and increased awareness of participation. (8) Integrates theoretical knowledge with practical skills, to develop a profound comprehension of the case. (9) Improved the ability to search and utilize literature. The students could choose one of three options—“strongly agree,” “partially agree,” or “don’t agree” for the above statements.

#### Insights and sentiments of the students about the case

Upon completion of the course, the students documented their individual learning experiences, insights and valuable recommendations for the teacher. The teacher diligently compiled and organized the feedback of the students, which served as a valuable teaching material for the future teaching sessions and as a reference for further improvements in teaching methods.

#### Practice reports

The students engaged in evidence-based practice throughout their clinical internships, focusing on specific clinical cases, and then submitting their practice reports. The evaluation criteria for the practice report encompassed evaluating its thorough incorporation of the five steps of evidence-based clinical practice, as well as its demonstration of independent learning and problem-solving skills. The five steps of evidence-based clinical practice include: (1) posing a clinical question, (2) searching for evidence, (3) evaluating evidence, (4) applying evidence, and (5) evaluating outcomes, which involves tracking and reassessing the implementation [9, 10].

### Data processing and analysis

The questionnaires were coded and entered into a computer at the end of the course. The data were statistically described as the composition ratio (%) using SPSS 13.0 software.

## Results

### Questionnaire

After completing the course, the students evaluated the effectiveness of the “autonomy-collaboration” learning modality through the utilization of evidence-based practice. This evaluation was conducted by means of a questionnaire, which was filled by the students independently and submitted immediately. A total of 91 questionnaires were received, all of which were valid, resulting in a validity rate of 100%. There were 28 males and 63 females, with an average age of 23.56 ± 0.81 years. Among the respondents, 65 students (accounting for 71.43%) expressed a partial agreement, whereas 26 students expressed complete agreement that the EBM course enhanced their medical knowledge, engaged their enthusiasm for learning, and improved their self-directed learning capabilities. Out of the total participants, 62 students (accounting for 68.13%) expressed a partial agreement, while 29 students completely agreed that the EBM course was a comprehensive discipline that integrated multiple disciplines and could improve the ability to utilize the various courses in a comprehensive manner and the ability to work in a team. In addition, 60 students were more likely to recognize that this modality improved the ability to review and utilize literature, while 57 students were more inclined to recognize the significance of EBM in making medical decisions. The majority of the participants felt that this modality of learning improved their skills to identify and raise questions in clinical practice. By utilizing this method of learning, they acknowledged the importance of compiling and reviewing information, as well as the necessity to find supporting evidence for any decision, thereby enhancing the sense of involvement. There was also feedback about the enhanced communication between teacher and students, thereby improving the ability to analyze and solve problems. See Table 1 for details.

### Ideas and feelings

Based on the insights and sentiments documented by the students, some students provided feedback on something they had not encountered in textbooks previously, namely the cognitive approach introduced by EBM. Some students observed that this learning modality not only improved their autonomy in learning but has also allowed them to comprehend the spirit of “practicing medicine for the people and achieving medical comprehension.” Many students also observed that learning EBM guided them towards better serving their patients. The integration of EBM theory with practical application deepened their understanding of medical knowledge and was beneficial in their clinical work. They also noted that this learning modality vastly aided them in writing academic papers and conducting scientific research. These records enabled the teachers to appreciate the significance of this teaching modality.

### Evidence-based clinical practice report

During the clinical internship stage, every student submitted an evidence-based practice report, grounded in the cases encountered in clinical practice and employing the principles of acquired EBM knowledge. The teacher discovered that the cases students encountered in actual clinical practice encompassed all types of clinical situations such as etiology, diagnosis, treatment, and prognosis. The students were meticulous in every step of their research process, from raising questions and identifying key terms, to search strategies and retrieving literature. A more detailed process is as follows: From the aspect of problem construction: In order to clarify the nature of clinical problems and facilitate retrieval, clinical problems must be reconstructed and transformed according to the“PICO” principle, for example, for clinical problems of etiology, “P” is the patient or population, “I” is the intervention or exposure factor, “C” is the control or placebo, “O” is the outcome indicator, such as fracture or incidence. In terms of determining keywords, search terms are often extracted from “PICO 4” elements and matched to form search strategies. If necessary, include the type of clinical question being asked and the type of evidence being sought. Literature search: First, Summaries are searched in the database, such as Best Practice, UpTo-Date, etc., depending on the subscription status of your organization. If the evidence in the Summaries database does not solve the problem, or the evidence included in the Summaries resource is of poor quality or is too old, then consider searching the non-summaries database. Clinical application: The quality of the retrieved literature is evaluated, and the authenticity, importance and applicability of the literature are satisfied in all three aspects, and the will and values of the patients are respected, and finally applied to the patients to solve clinical problems. As a result, their practical reports were executed proficiently. The students expressed that the exposure to the actual cases deepened their understanding of the theoretical knowledge in the classroom.

**Table 1:** Evaluation of the effectiveness of the “autonomy-collaboration” learning modality based on evidence-based practice

	Comparative Recognition		Recognized		Disapprove	
Items	Number (persons)	Composition ratio (%)	Number (persons)	Composition ratio (%)	Number (persons)	Composition ratio (%)
1. The course of study enhanced medical knowledge, stimulated learning motivation, and improved learning autonomy	65	71.43	26	28.57	0	0.00
2. The evidence-based medicine course is a comprehensive subject that integrates multiple disciplines and improves the comprehensive utilization of various courses and collaborative skills.	62	68.13	29	31.87	0	0.00
3. Acknowledge the importance of evidence-based medicine in making medical decisions.	57	62.64	33	36.26	1	0.01
4. Improve the ability to identify and raise questions in clinical practice.	50	54.95	41	45.05	0	0.00
5. Adeptly and accurately identifying the type of issue is critical before proceeding to other steps.	58	63.74	32	35.16	1	0.01
6. Acknowledge the significance of accessing and compiling information.	59	64.84	32	35.16	0	0.00
7. Obtain supporting evidence for any decision and increase awareness of participation.	51	56.04	39	42.86	1	0.01
8. Integrate theoretical knowledge with practical skills, to develop a profound comprehension of the case.	55	60.44	36	39.56	0	0.00
9. Improve the ability to research and utilize literature.	60	65.93	31	34.07	0	0.00

## Discussions

The application of the “autonomy-collaboration” learning modality based on evidence-based practice for the 2019 batch of clinical medicine students at Zhongshan Clinical College of Dalian University yielded positive outcomes. Among the respondents, 65 students (accounting for 71.43%) expressed partial agreement, while 26 students completely agreed that the evidence-based course enhanced their medical knowledge, engaged their enthusiasm for learning, and improved their self-directed learning capabilities. Among all the respondents, 62 students (accounting for 68.13%) partially agreed, while 29 students completely agreed that the evidence-based course was a comprehensive subject that integrated multiple disciplines and could improve the ability to utilize the various courses in a comprehensive manner and the ability to work in a team. Furthermore, 60 students highly appreciated this modality for its effectiveness in improving literature search and utilization skills. Additionally, 57 students highlighted the significance of EBM in making healthcare related decisions. This is attributable to their engagement in the problem-solving process, wherein they translate theoretical knowledge from textbooks into various practical skills. Consequently, a comprehensive, multidisciplinary, and interconnected knowledge network is established over time. Additionally, there was feedback indicating that communication between teachers and students was strengthened, leading to improvements in analytical and problem-solving skills. The significance of EBM in medical decision-making was appreciated, and the sense of involvement was enhanced. In this modality, the teacher placed emphasis on fostering students’ skill development, cultivating their proactive learning, and enhancing their capacity for information retrieval. Furthermore, the integration of EBM knowledge during internships and practical stages contributes to the establishment of a comprehensive understanding of EBM among medical students. This integration enhances students’ proficiency in utilizing literature information and fosters a heightened interest in learning. Consequently, it successfully attains the objective of enhancing the medical information knowledge of the students [2, 11]. Various clinical scenarios were created in the classroom to maximize the motivation and initiative abilities of the students. Facilitating effective guidance for students to engage in independent learning and collaborative group learning, coupled with encouraging their confidence in expressing insights and perspectives, proved conducive to the enhancement of students’ analytical skills.

In summary, the “autonomy-collaboration” learning modality based on evidence-based practice, allows medical students to take full control of their learning process, and enhances their development of a proactive attitude toward seeking knowledge and conducting innovative research. It has changed the previous passive modality of learning [12]. This modality has transitioned from a single classroom theoretical lecture to a blended learning approach that combines relevant theoretical lectures with practical case studies. The teaching method allows students to proactively engage in learning based on the problems presented in the cases and pursue independent learning under the guidance of teachers. Based on this modality, students communicate with each other to discuss common issues together and promote the development of their autonomy and collaborative abilities [13]. This modality adopts a “autonomy-collaboration” teaching method in the classroom, with supplementary clinical practice outside the classroom. It combines theoretical knowledge and practical skills built upon the foundation of conventional learning. Through clinical practice, it promotes the development of innovative spirit and abilities and contributes to comprehensive improvements in the quality of teaching. In the contemporary landscape of undergraduate medical education, this instructional approach is centered on students, with clinical practice serving as the foundational cornerstone. It employs evidence-based principles and methodologies to structure teaching activities, guiding students to critically evaluate traditional teaching concepts and instilling a mindset of continuous learning. The ongoing updating of knowledge within this methodology bears significant practical relevance. The learning model of “autonomy-collaboration” based on evidence-based practice has been well applied in teaching, promoting the integration of theory and clinical practice.

In summary, this teaching model changed traditional teaching methods, effectively trained students‘ clinical thinking, and cultivated their ability to identify, analyze, and solve problems. It has improved the quality of EMB education. However, there are also limitations, such as the limited number of recruited students, which were at the same grade. In addition, direct quotations from students and teachers were not recorded. Further investigations are needed to expand the scope of research subjects, quantify practical reports, and evaluate various teaching effects to reflect the effectiveness of this teaching model.

## Data Availability

The datasets used and/or analysed during the current study available from the corresponding author on reasonable request.
